# Nanomaterials for Wound Dressings: An Up-to-Date Overview

**DOI:** 10.3390/molecules25112699

**Published:** 2020-06-10

**Authors:** Alexandra Elena Stoica, Cristina Chircov, Alexandru Mihai Grumezescu

**Affiliations:** Department of Science and Engineering of Oxide Materials and Nanomaterials, Faculty of Applied Chemistry and Materials Science, University Politehnica of Bucharest, 1-7 Gheorghe Polizu Street, 011061 Bucharest, Romania; elena_oprea_93@yahoo.co.uk (A.E.S.); cristina.chircov@yahoo.com (C.C.)

**Keywords:** wound dressings, nanomaterials, bionanomaterial, nanotechnology, nanomedicine, semi-permeable films, semipermeable foam dressings, hydrogel dressings, hydrocolloid dressings, alginates dressings, non-adherent contact layer dressings, multilayer dressings

## Abstract

As wound healing continues to be a challenge for the medical field, wound management has become an essential factor for healthcare systems. Nanotechnology is a domain that could provide different new approaches concerning regenerative medicine. It is worth mentioning the importance of nanoparticles, which, when embedded in biomaterials, can induce specific properties that make them of interest in applications as materials for wound dressings. In the last years, nano research has taken steps to develop molecular engineering strategies for different self-assembling biocompatible nanoparticles. It is well-known that nanomaterials can improve burn treatment and also the delayed wound healing process. In this review, the first-line of bioactive nanomaterials-based dressing categories frequently applied in clinical practice, including semi-permeable films, semipermeable foam dressings, hydrogel dressings, hydrocolloid dressings, alginate dressings, non-adherent contact layer dressings, and multilayer dressings will be discussed. Additionally, this review will highlight the lack of high-quality evidence and the necessity for future advanced trials because current wound healing therapies generally fail to provide an excellent clinical outcome, either structurally or functionally. The use of nanomaterials in wound management represents a unique tool that can be specifically designed to closely reflect the underlying physiological processes in tissue repair.

## 1. Introduction

**Figure 1:**
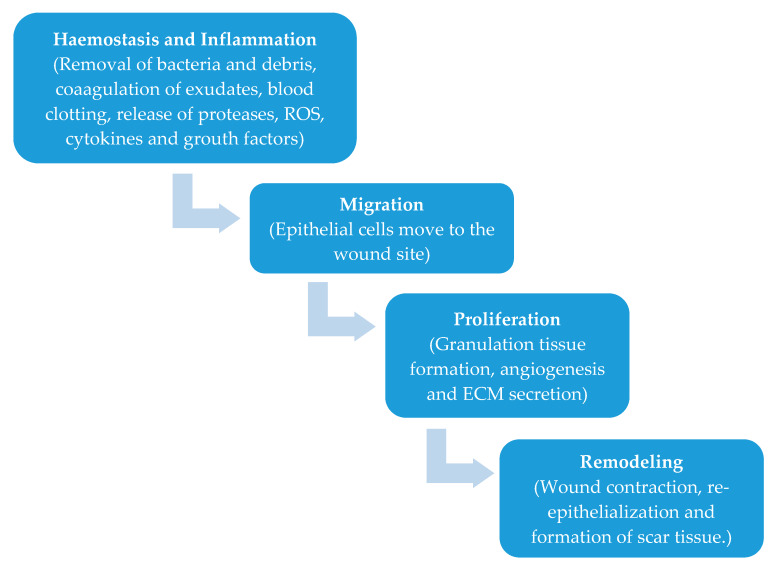
Phases of wound healing [2]. Reprinted from an open-access source.

The phases involved in the wound healing process are hemostasis, inflammation, proliferation, and remodeling (Figure 1) [1,2]. Wound healing represents a very complex process that requires a long time to be complete [3], as the remodeling phase to form the proper environment can last from 21 days to 1 year [4,5]. 

Wound management is an ongoing process that involves the complete spectrum of holistic clinical measures, methods, and interventions in the care of patients with wounds [6,7]. Appropriate wound care management represents a significant clinical challenge, and there is an expanding necessity for the wound care domain [8]. Chronic wound care represents an area of interest in the research domain by developing new and efficient materials for wound dressings [9,10,11]. Concerning how the decisions regarding dressing product selection are made, doctors must guarantee an assessment of the patient’s suffering and wound etiology [12]. It is also essential to know and understand the dressing features when making clinical choices by adopting wound management protocols to provide optimal innate reactions [13,14,15].

For an efficient treatment, the choice of the wound dressing must consider various important wound-associated aspects, including the type and profoundness of the wound and also the amount of exudates [16,17,18,19,20]. An ideal wound dressing is the one capable of maintaining or ensuring a moist environment required for the healing process [21,22,23,24]. Moreover, the dressing should tolerate proper gas permeability [25,26] and extract the excess exudate from the wound surface while maintaining it moist [27,28]. Additionally, another important aspect is related to the ability to act as a barrier for microorganisms by providing an antibacterial medium [29,30,31]. It is imperative to mention that the dressing must be biocompatible, low-cost, and easy-to-apply, without requiring constant replacement [4,18,32,33,34,35,36]. 

**Figure 2:**
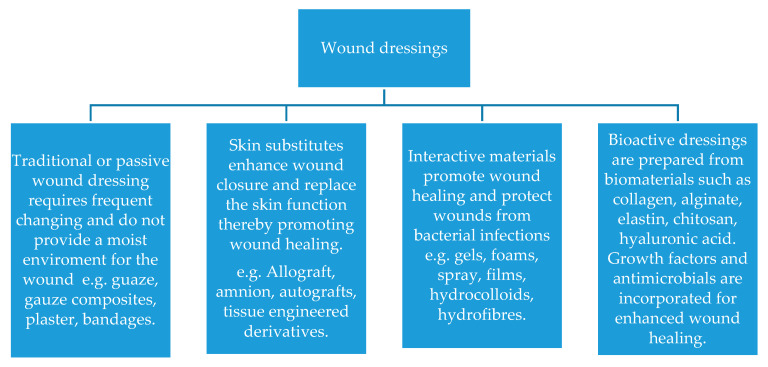
Classification of wound dressings [2]. Reprinted from an open-access source.

Over the past few years, the research and development field concerning wound dressing materials has installed an advanced stage of standards, which seems to have led to an adequate understanding regarding the pathogenesis of chronic wounds. Different types of dressing are utilized for wound healing, and they can be classified based on different factors. First, dressings can be categorized in three types, namely: (1) traditional dressings (e.g., a gauze and gauze/cotton composites) (2) biomaterial-based dressings (allografts, tissue derivatives, and xenografts) and (3) artificial dressings (film, membrane, foam, gel, composites, and spray) [37,38,39,40]. Second, wound dressings are classified into two categories: (1) primary dressings – applied directly on the wound; (2) secondary dressings – used to cover the primary dressing [41,42,43]. Furthermore, based on the interaction with the biological tissue, dressings can be classified as (1) passive/inert dressings [44,45] and (2) interactive/bioactive dressings [46,47] (Figure 2). Regarding the first-line interactive/bioactive dressings, they are immediately available for application in acute and subacute wounds [48]. Concerning the second-line dressings, these are unconventional and also include the antimicrobial dressings category [41].

This review aims to present the physical and biological properties, the forms of the dressings, the advantages and disadvantages, and the indications and contraindications of using the first-line interactive/bioactive dressing class based on nanomaterials frequently applied nowadays in clinical practice. These include the application of nanomaterials for semi-permeable films, semipermeable foam dressings, hydrogel dressings, hydrocolloid dressings, alginate dressings, non-adherent contact layer dressings, and multilayer dressings. Additionally, this review aims to highlight the lack of high-quality evidence and the necessity for future advanced designed trials because current wound healing therapies generally fail to provide an excellent clinical outcome, either structurally or functionally. As a result, various combinations of both natural and synthetic materials have been developed: films, sponges, hydrocolloids, hydrogels, fiber mats etc. However, nanomaterials-based treatments have achieved a new horizon in the arena of wound care due to their ability to deliver a plethora of therapeutics into the target site and to target the complexity of the normal wound-healing process [49,50].

## 2. Nanomaterials-Based Wound Dressing

Conventional wound dressing materials can produce wound dehydration. They can mechanically attach onto the wound surface, which can make the replacement of the wound dressing an uncomfortable and even painful process [3,51,52,53,54]. In this manner, wound-healing therapy based on the utilization of nanomaterials (NMs) has provided new approaches and benefits in this field [40]. At present, there are two types of NMs for wound-healing therapy that can be discussed. Specifically, nanomaterials which are capable of healing as a result of the inherent properties of the nanoscaled material and nanomaterials as carriers for delivering therapeutic agents. The effects of various nanomaterials and their ability of wound healing are dissimilar and mostly depend on the NMs physicochemical properties [40,55]. Some essential characteristics of NMs, which may influence the effect of the wound healing process, are biocompatibility and biodegradability, colloidal stability, size, surface functionalization, and surface charge. The biodegradability and biocompatibility of nanomaterials has supplementary benefits over particles that cannot be digested and deposited within the body. However, more advantageous than the physicochemical properties is the presence of a payload (active ingredient) with wound-healing activity within NMs [40,56,57].

**Figure 3:**
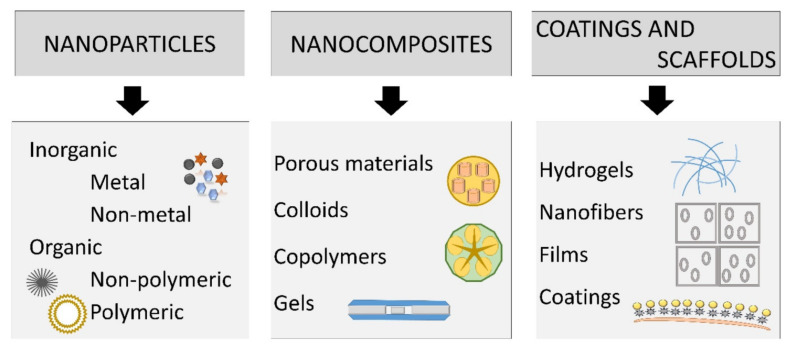
Main types of nanomaterials can be used for wound treatment [58]. Reprinted from an open-access source.

NMs, owing to their particular properties, open a new direction of wound-healing products. NMs can change each phase of wound healing as they have antibacterial, anti-inflammatory, proangiogenic, and proliferative properties. Moreover, NMs are capable of modulating the expression level of different essential proteins and signal molecules in order to improve the wound healing process. Thus, NMs or the combination of materials at both micro- and nanoscales may become favorable enough to overcome most of the challenges that exist in wound care management [40]. The main types of nanomaterials which can be used for wound treatment are nanoparticles, nanocomposites, and coatings, and scaffolds (see Figure 3) [58].

The use of natural and artificial 3D architectures as scaffolds for support, with or without cells, is one of the most applied strategies in tissue engineering. Nanotechnology furnishes excellent tools for the introduction of nanotopographic signaling in biomaterials that can adequately mimic the extracellular matrix (ECM) and the cellular microenvironment [3,59,60]. Modifying the surface and 3D structure at the nanoscale is feasible, and NMs may be combined within a larger guiding template or scaffold [3,59]. As the microenvironment is critical to wound healing, it is crucial that any cell-based therapy supports new or host cell populations by providing a suitable microenvironment [60,61]. 

Thus, by contrast to the disadvantages of traditional wound dressings, it is essential to design a novel wound dressing that does not lead to further injury, presents good antibacterial effects, and promotes wound healing [62].

### 2.1. Semipermeable Film Dressings

Semipermeable film dressings are flexible, thin, and transparent, sheets of polyurethane or copolyester covered with an adhesive layer that allows the dressing to adhere to the skin [63,64]. Film dressings furnish a protective environment that is impermeable to bacteria and liquids but permeable to water vapors, O_2_, and CO_2_. They can remain in place for one week [65]. Semipermeable film dressings are designed for superficial dry wounds, such as simple abrasions, minor burns, or lacerations [41,66]. These can be used as primary dressings or as secondary dressings when applied at the same time with foam dressings for severe exuding wounds [67]. Also, they are used quite often as postoperative films over dry sutured wounds [68]. Recent approaches suggest that the application of surgical film dressings obtained from polyurethane in order to treat postoperative wounds are more efficient and cost-effective when compared to well-known traditional gauze dressings [69]. Owing to the advantage of being transparent and the capacity to be molded to the body shape (such as elbows, knees, sacral areas) due to their flexible and elastic nature [2,70], semipermeable film dressings can be easily applied on different locations of the wound. While island dressings that contain a central non-stick pad can absorb slightly more exudate, semipermeable film dressings are not appropriate for exuding wounds because they have a non-absorbent character [41,69].

However, film dressings must be carefully removed because their adhesiveness can lead to damage of the epidermal skin layer [71,72]. Consequently, inappropriate removal of film dressings, when used to treat delicate skin, can provoke an unwanted trauma to the surrounding skin [73]. Semipermeable film dressing should be discontinued when the level of exudate results in pooling under the dressing [38]. Semipermeable films are non-absorptive dressings, and inappropriate dressing choices can lead to the maceration of the surrounding skin and, at the same time, to the increase of the infection risk. Films can be maintained in place for up to seven days, and the replacement period may depend on the size and type of the wound and also on its location [41].

New approaches regarding nanomaterials-based film dressing are currently described in the literature. Hubner et al. [74] recently described novel gelatin-based films using glycerol as a plasticizer and incorporated with different concentrations of clinoptilolite zeolite impregnated with silver ions as wound dressings. Because dressings applied in both acute and chronic wound treatment must present antimicrobial characteristics, silver-based compounds were used as antiseptics (Figure 3). For this purpose, films were produced by casting. All tested concentrations of gelatin/ clinoptilolite-Ag films presented antibacterial activity against *S. aureus* and human skin bacteria, not presenting meaningful differences in the size of the formed halo.

**Figure 4:**
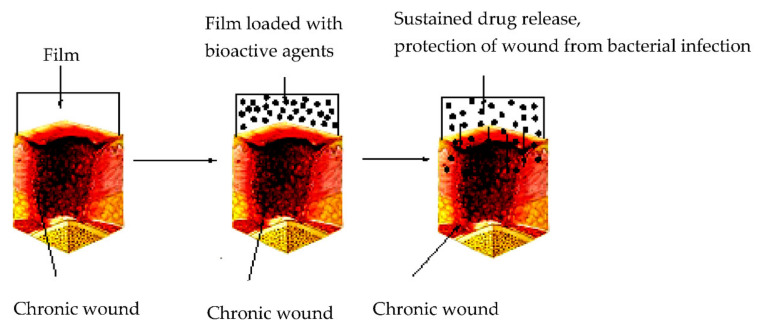
Films for wound dressing [2]. Reprinted from an open-access source.

The electrospinning technique can also be used to prepare nanofiber-based semipermeable film dressings [75,76,77,78,79,80]. In this regard, nano-fibrous membranes as smart wound dressings that release antibiotics when an injury is infected were obtained by Rivero et al. [75]. Thus, electrospun film dressings capable of releasing nitrofurazone when there is a variation in the pH of the environment were successfully prepared using polymers with selective solubility at pH values greater than 7. This study proved the importance of nanotechnology in the medical field. These pH-sensitive nano-fibrous drug delivery carriers were recommended as intelligent wound dressings with notable potential for improving the therapeutic advantages during medical treatments, according to the physiological conditions of the damaged skin tissues (Figure 4). Beyond the passive role of conventional wound dressings, the nanostructure of electrospun films is ideal for use in this application. Therefore, electrospinning is widely used to fabricate nanoscale fibers from natural and synthetic polymers [81] and nanomaterials-based film dressings using different complex mixtures of polymers and nanoparticles, such as cerium oxide nanoparticle-containing poly(ε-caprolactone)/gelatin [82], polymer-titanium dioxide nanocomposites [81], silver nanoparticle and riboflavin loaded PVA/β-CD [83], polyvinyl alcohol/chitosan nanofiber with carboxymethyl chitosan nanoparticles encapsulating the antibacterial peptide OH-CATH30 [84], carboxyethyl chitosan/poly(vinyl alcohol)/silk fibroin nanoparticles [85], honey loaded alginate/PVA [86], or polyvinyl alcohol/starch/glycerol/citric acid [87].

Furthermore, Ambrogi et al. [88] studied two different alginate films containing pyrogenic silica-supported silver nanoparticles, which proved excellent hydration properties and a prolonged silver nanoparticles release, as potential wound dressings. The obtained films showed antimicrobial and antibiofilm activities against *S. aureus* and *P. aeruginosa*. They displayed no cytotoxicity towards human fibroblasts HuDe and human skin keratinocytes. An essential aspect of wound healing is represented by the ability of wound dressing materials to control drug release. Mazloom-Jalali et al. [89] studied these aspects by developing biocompatible nanocomposite films based on chitosan and polyethylene glycol polymers containing cephalexin antibiotic drug and zeolitic imidazolate framework-8 (ZIF-8) nanoparticles. The tests on these materials showed antibacterial activities against different bacteria (especially against the *Bacillus cereus* bacterium), and all films had high cell viabilities to L929 fibroblast cells. Among the modern wound dressings, semipermeable film dressings are considered to be one of the significant advances in wound management [90].

### 2.2. Semi-Permeable Foam Dressings

Foam dressings are generally obtained from porous polyurethane foam or polyaniline sponge-like polymer with a semi-occlusive hydrophobic backing [63]. These kinds of dressings are absorbent, and the absorption property can be controlled through the foam’s thickness, texture, and pore size [71]. The semipermeable foam dressings present both gas and water vapor permeability but are impermeable to bacteria and fluids [63]. As a result of the porous structure of the dressings, they could be applied for partial- or full-thickness wounds with slight or medium drainage, heavily exuding slough-covered or granulating wounds, and also graft donor sites [63,71]. Some studies confirmed their beneficial effect on granulating wounds, reporting that semipermeable foam dressings help treat granulation when applied with slight pressure. These classes of dressings preserve a moist environment, produce thermal insulation, and are comfortable to wear [91]. Semipermeable foam dressings could be applied as a primary dressing, or on top of hydrogels or creams acting as secondary dressings [92]. Foam dressings are not appropriate for dry scars or dry epithelializing wounds when they depend on exudates in order to ensure a beneficial environment for wound healing [71]. Foam wound cavity dressings reduce necrotic tissue in the wound, adapt to the wound form, and absorb large amounts of exudate, thus decreasing the need for frequent dressing replacements. However, because foams are, in most cases, non-adhesive, a secondary dressing or tape/bandage is necessary to keep it in place, which is further associated with additional costs. [41]. The semipermeable foam dressings should be changed once saturated with exudate; this can range from daily to once or twice a week [63].

Polyurethane-based semipermeable dressing foams have been extensively used due to their excellent water absorption capability, optimal mechanical properties, and economic advantages (Table 1). Nonetheless, the low bioactivity and poor healing ability of polyurethane reduce the applications of polyurethane foam dressings in complex wound healing cases [93,94]. In order to overcome this problem, different nanomaterials were widely investigated by researchers. A recent study published in 2019 by Bužarovska et al. [95] showed the ability to enhance the antimicrobial activity of a foam dressing by adding different content of ZnO nanofiller. The induced phase separation method was used to fabricate biobased thermoplastic polyurethane/ZnO nanocomposite foams. Their results proved that the obtained foams dressings possess appropriate morphology to keep the proper environment at the wound/dressing interface. Further, dressings showed low cytotoxic potential and were very effective against *Gram-positive* and *Gram-negative* bacteria. Moreover, one performed a comparative study between the efficacy of silver nanoparticle gels, nanosilver foams, and collagen dressings in partial-thickness burn wounds [96,97]. 

The efficiency of the dressings was evaluated in terms of healing rates, time and ease of application, pain at dressing removal, wound-swab culture, scar quality (at 3 months), and costs. Nanosilver-foam dressings were found to be more efficient for healing, re-epithelialization, ease of application, tolerance when compared to collagen dressings, and silver nanoparticle gel dressing in partial-thickness burns. It is essential to mention that silver nanoparticles had an essential role regarding the healing and re-epithelialization process since their antimicrobial activity.

Silver nanoparticles were also used by Chen et al. [97] to obtain composite sponges with great potential in promoting wound healing based on Konjac glucomannan. The Konjac glucomannan/silver nanoparticle composite sponge showed great water absorption and retention and essential mechanical properties. Owing to the presence of silver nanoparticles, sponges showed good antibacterial activity against test microorganisms. In animal models, they promoted fibroblast growth and accelerated epithelialization.

Polyurethane-silica hybrid foams, which have significantly enhanced biocompatibility and wound healing capabilities compared to pure polyurethane foams, were successfully fabricated by Song et at. [93]. They used the hybridization of bioactive silica nanoparticles with polyurethane through a one-step foaming reaction that is coupled with the sol-gel process. It was clearly proved that polyurethane-silica hybrid foams present significant potential as wound dressing material geared for accelerated, superior wound healing. Even though foam dressings are indicated for the management of venous ulcers with moderate and high exudate levels, their efficacy is yet to be reported. There was no clear evidence that semipermeable foam dressings are generally more efficient than other dressings applied for the management of diabetic foot ulcers [41].

### 2.3. Hydrogel Dressings

Wound healing is a complex and dynamic process and involves a series of events, which create a unique microenvironment at the wound sites. It is highly desirable to develop multifunctional skin substitutes, which can play their roles in the whole healing process to enhance the final healing efficiency [101]. Hydrogels have many advantageous properties, such as softness and easy water retention [102,103,104]. These properties prevent tissue dehydration, and for that reason, hydrogels can be applied in the preparation of dressings for acute/chronic wounds, burns, and also diabetic foot ulcers [37,105,106,107,108]. Moreover, hydrogels are insoluble, swellable polymers which have a high water content, and they come in the form of an amorphous gel, or as an elastic solid sheet or film [103,109]. This kind of dressings is perfect for supporting autolytic debridement of necrosis and slough by rehydrating dead tissues [110]. Hydrogels are semi-transparent and semipermeable to gases and fluids [63]. Hydrogels are non-adherent dressings that stimulate healing and cool the surface of the wound by up to 5 °C, leading to a decrease of pain [111,112]. Furthermore, hydrogel dressings do not leave residues and promote wound re-epithelialization. In terms of the hydrogel, a secondary dressing is necessary, which does not influence the ability of the hydrogel to offer water to the wound bed [113]. The hydrogel sheets could be cut to fit around the wound owing to their flexible nature. As a result of their considerable amount of water (70–90%), hydrogel dressings are not able to absorb much exudate and are, consequently, recommended for dry and minimally exuding wounds. The fluid accumulation may lead to skin maceration and bacterial proliferation. It can cause wound infection and also a foul smell [114]. Further, hydrogels have low mechanical strength and are, therefore, difficult to handle [71]. Hydrogels should be changed generally every 1–3 days, depending on the wound hydration status [115]. Care must be taken to ensure the dressing replacement is frequent enough to prevent maceration of the surrounding skin [63].

In order to improve the healing properties and antimicrobial activity of the hydrogel dressings, different nanomaterials were added in the composition of the dressings. Silver nanoparticles seem to be again a promising alternative [116,117]. Nešović et al. [116] provided an efficient method for the development of wound dressing materials with enhanced properties based on biocompatible chitosan and poly(vinyl alcohol) hydrogels with embedded silver nanoparticles as a potent antimicrobial agent. The hydrogel dressings were confirmed to be non-cytotoxic and possessed significant antibacterial activity against *S. aureus* and *E. coli*. In the treatment of chronic wounds, pressure ulcers become a particular challenge because the main method of treatment is to prevent infection while allowing the wound to heal [117]. The silver nanoparticles-based PVP/alginate/chitosan hydrogel in the ratio of 10:1.2:1.8 showed antibacterial properties, no cytotoxicity, reduced cost (compared to commercial ones, e.g., Algivon^®^, ACTICOAT™, and Suprasorb^®^ A + Ag), and maximum swelling. 

Antibacterial efficiency of ZnO nanoparticles has also been explored in order to produce superior hydrogel wound dressings [118,119]. Khorasani et al. [119] prepared a tested heparinized poly(vinyl alcohol)/chitosan/nano zinc oxide hydrogels. They proved that by adding nano zinc oxide, the mechanical and thermal properties improved while the heparin release rate decreased. The antibacterial character of the hydrogel obtained can effectively protect wounds, especially with an increased nano ZnO amount. Additionally, xanthan-poly(vinyl alcohol)/ZnO nanocomposite hydrogels have been obtained by Raafat et al. [118] as active wound dressing using Gamma irradiation as an eco-friendly source for cross-linking and sterilization processes. The obtained hydrogel dressings proved an effective microbial barrier potency and important antimicrobial activity against *S. aureus, E. coli,* and *C. albicans*. Also, in vitro cytotoxicity and hemolytic potency assessment demonstrated their biocompatibility. A similar technique of hydrogel dressings preparation was used by Boonkaew et al. [120] in 2014, which proposed a dressing containing silver nanoparticles to treat infection in a 2-acrylamido-2-methylpropane sulfonic acid sodium salt hydrogel. The results also showed in this case that the prepared dressings are nontoxic and have good inhibitory action against *P. aeruginosa* and methicillin-resistant *S. aureus*.

Liposomal hydrogel as a wound dressing furnishes a barrier that prevents bacterial contamination of the wound and further evolution of infection to the deeper tissues [121]. Nunes et al. [122] demonstrated that collagen-based films containing liposome-loaded usnic acid are useful in improving burn healing. Shailesh and Kulkarni [123] developed and evaluated a liposomal hydrogel of mupirocin as a diabetic wound dressing. They proved that this type of dressing combines the beneficial properties of both sustained releases of the drug in preventing infection and moist wound dressing with good fluid absorbance. Another study published by Değim et al. [124] presents the production of a chitosan gel formulation containing liposomes loaded with epidermal growth factor in order to evaluate their effects on the healing of second-degree burn wounds in rats. The histochemical results showed significant increases in cell proliferation and an improved epithelisation rate. 

The main advantage of hydrogels is represented by the creation of a moist and cool environment for wound healing and supporting high water vapor permeability along with avoiding penetration of microbes into the wound surface [119].

### 2.4. Hydrocolloid Dressing

Hydrocolloid dressings are fabricated from colloidal, gel-forming materials, mixed with elastomers and adhesives [125]. Hydrocolloid sheet dressings consist of two main layers. The inner layer is a self-adhesive layer composed of a hydrophilic polymer matrix with dispersed gelatin, pectin, and other substances [126]. The outer layer generally consists of polyurethane, and it protects the wound from bacteria, foreign debris, and shear forces [63]. Usual hydrocolloid dressings include carboxymethyl cellulose, gelatin, and pectin. These dressings appear in the shape of thin films and sheets, or as composite dressings combined with other various materials, and are applied for light to moderately exuding wounds [71]. Hydrocolloid dressings are also attainable in powders and pastes [63]. After exudate absorption, a modification in the physical state occurs because of the gel formation. The most important aspect is represented by the painless dressing removal and also the possibility to use them in pediatric wound care management of acute and chronic wounds [108,127]. That is probably one of the main reasons why hydrocolloid dressings are the most widely used dressings [63,128]. Hydrocolloid wound dressings, obtained from a hydrophobic pressure sensitive adhesive (the continuous phase) and hydrophilic filler (the dispersed phase), can heal wounds faster with less pain [129,130]. At the same time, patients may do regular activities without causing wound damage [131].

Hydrocolloid dressings are semi-permeable to water and gas vapors but impermeable to fluids or bacteria. This type of dressings, in most cases, avoid water vapor exchange, which is a drawback for applying in infected wounds that need a specific amount of oxygen for an improved healing process [71]. Hydrocolloids are waterproof and cushioned and do not need a secondary dressing, so they are advantageous to use [132]. Hydrocolloid dressings should be maintained in place until the drainage is observed beneath the dressing; daily replacements should be performed early in the treatment course, with a decrease to every three days to once a week over time [63,133].

Hydrocolloids are regularly applied in pressure ulcer treatment [134,135,136,137]. For this application, hydrocolloid dressings are more efficient than gauze dressings owing to the number of healed wounds and the decreased pressure ulcer dimensions [138,139]. Also, a study published in 2018 by Halim et al. [140] concluded that chitosan derivative film is equivalent to hydrocolloid dressing and can be an option in the management of superficial and abrasion wounds. It seems that hydrocolloid dressing was also evaluated for its potential application for neurosurgical wounds according to the modern concept of wound healing [141]. Clinical evaluations were realized for wound infection, wound healing, and cost-effectiveness and proved excellent wound healing and cosmetic results.

Development of new biocompatible and biodegradable hydrocolloid dressings based on nanomaterials, which could be able to sustain all phases of wound healing, should be a future perspective for researchers due to the lack of publications in this area.

### 2.5. Alginate Dressings

Alginate is the most widely used ionic polysaccharide applied in the design and development of various wound dressing materials -enhancing the efficacy of the wound healing process [142]. Taking into account the various medical requirements, alginate is applied in disparate derivative materials. Its derivative materials, including wafers [101,102,103,104], foams [2,105,106], gauzes [107,108,109], and fibers [110,111,112] are widely used for wound healing [44,113]. Among these, nanofibers and microfibers have their particular interest in clinical applications. The nanofibers and microfibers can have very high porosity for the exchanges of wound gases and fluids [112]. Further, appropriate pores (1–10 µm) are required to prevent bacteria from the atmospheres and large surface area to absorb exudates and antibiotics [44,112].

Most of all, alginate enhances the hydrophilic character of wound dressing materials to create the necessary moist wound environment [51,143], extract wound exudate, and improve the ability of skin healing of the wound [42,144]. Further, alginate should easily cross-link with various organic or inorganic materials, and they can promote wound healing in clinical applications [44,114]. This is as a result of bioactive alginate that may enhance the hydrogel properties (e.g., optimal moisture vapor-transmission, biodegradability, exudate absorption) with their intrinsic swelling characteristics [44,115]. All of these are favorable for wound healing and are more valuable as sterile dressing materials for hemostatic applications as well as secondary applications [42,51].

Alginate dressings are obtained from soft, nonwoven alginic acid fibers, a cellulose-like polysaccharide derived from seaweed, coated in calcium and sodium salts [63]. They have the capacity to form gels at the time of contact with wound exudates, which permits easier dressing removal [145]. Alginate dressings are indicated for moderate to heavily exuding wounds. The strong hydrophilic gel formation induces the high absorption properties, which restrict wound secretions and reduce the bacterial contamination to a minimum [144,146]. Gel formation is a result of Ca^2+^ ion exchange from alginate fiber with Na^+^ in exudate. This aids in maintaining favorable conditions for wound healing, such as an optimum moisture amount and healing temperature.

Nevertheless, released Ca^2+^ into the wound is helping the clotting mechanism in the course of the first stage of wound healing [147]. Alginate dressings are very absorbent (15–20 times their weight in fluid), biodegradable, and non-adherent [63,148]. Since alginates need moisture to act appropriately, they cannot be applied for dry wound treatment or wounds with necrotic tissue as they could dehydrate the wound and delay the healing process [71,148]. The alginate dressings are the best dressing choice for highly exudative wounds [148]. Due to the Ca^2+^ released from the dressing activate prothrombin in the clotting cascade, they also are helpful for hemostasis [63]. Alginates need a secondary dressing and should be changed up to 1 week or until the gel loses its viscosity [144,148]. Because alginate dressings are soluble and could be removed by saline irrigation, their replacement is not painful. The yellow-brown color and malodorous smell of alginates could be incorrectly correlated with infection [63,148].

Various types of biopolymers have been used to fabricate alginate-based wound dressing materials [144,148] for the development of highly flexible, mechanically robust, and economically low-cost wound dressings [51,149,150]. As a result of their functional groups’ alginate can be efficiently mixed with different biopolymers and consequently generating cross-linked network structure. These cross-linked network structures could improve the physical stability of the wound dressing material and also furnish a moist wound environment [51,147].

Over the past few years, alginate-based foams (porous matrices) have attracted extensive interest in the field of wound dressing applications, considering their high surface area, high porosity, pore-volume, and water absorption capacity [88]. 

Wafers are utilized to apply, directly, the required quantity of therapeutic agents to the wound sites to decrease skin related problems, such as tissue maceration, and improve the wound healing properties [151,152]. Wound dressing wafers are principally produced from gel-forming polymers (which present the ability to absorb exudates slowly, e.g., alginate), by using the lyophilization method, which may create a solid matrix. It is essential to mention the 3D nature, porosity, and regular structure of the wafers may turn into a high viscosity product that can be comparable to foam. Nevertheless, wafers absorb wound fluids and transform into a gel which should provide essential moist environments for wound healing [51,153].

At present, significant effort is made to overwhelm bacterial infection and combat bacterial resistance. Regarding this context, the development of efficient and safe antimicrobial wound dressings which should selectively fight against the bacteria and reduce the disruption of healthy cells like red blood cells in wound bed is significant [153]. In a study published in 2020 by Maryam Zare-Gachi et al. [154], a series of ammonium salts of alginate were prepared, and the role of different counter-cations including sodium, triethylammonium, tributyl ammonium, and dihexylammonium were tested concerning antimicrobial efficiency and selectivity along with fibroblasts viability. *In vitro,* biological studies showed that tributyl ammonium alginate possesses optimum anti-hemolytic and antibacterial properties with less cytotoxicity at 1 mg/mL compared with different counter-cations. The histopathological analysis of tributylammonium alginate fibrous mat showed that this type of dressing improved re-epithelialization of infected full-thickness skin wounds together with the commercial silver-impregnated calcium alginate wound dressing

Despite the fact that alginate is an essential material for the production of wound dressings, there is also a necessity to choose proper reagents. These initiators should not be obtained by cross-linking that can induce toxicity and block the permeability of gases [144]. Alginate-based dressings are currently used clinically in wound healing applications. The development of future dressings containing bioactive agents is likely to play a much more influential role in the management of wounds [2,42]. Regarding all these characteristics, nanomaterials can be used as therapeutic delivery agents and help in wound healing [51]. Since the antimicrobial properties of cerium ions and chitosan are known, Kaygusuz et al. [155] combined the advantages of these materials. They reported for the first cerium cross-linked alginate-chitosan films. Samples showed proper water vapor transmission rate and notable antibacterial activity against *E. coli* and *S. aureus*. Another study published by Munhoz et al. [156] combined the silver sulfadiazine antimicrobial properties with a regenerative, biocompatible, and non-toxic character of alginate. Their results proved a suitable approach for obtaining innovative active wound dressings integrated into efficient drug delivery. Another method to prepare antimicrobial sodium alginate dressing was described by Liang et al. [157] in recent research published in 2020. Oxidized sodium alginate sponge was functionalized via polydopamine/silver composite nanospheres. Results proved low cell cytotoxicity, good blood compatibility, high hemostatic performance, and superior inhibitory activity against *S. aureus*, *P. aeruginosa,* and *E. coli*. It seems that the proposed method could open a novel way for antimicrobial sodium alginate dressing development. Alginate wound dressings based on nanomaterials are a promising approach in wound therapy, and improved materials can be obtained by combining the properties of alginate with the nanomaterials’ advantages.

### 2.6. Non-Adherent Contact Layer Dressings

Non-adherent contact layer dressings are obtained of a fine, woven mesh of polyethylene or polyethylene terephthalate that favors exudate to pass through. These dressings are applied directly onto the wound, providing an interface with the secondary absorbent dressing or pad [158,159]. A secondary pad or dressing is always needed. Non-adherent contact layer dressings are used for the preservation of newly formed tissue and stay in place for up to 2 weeks. However, the secondary dressing must be changed as frequently as required. Therefore, the disturbance of the wound bed healing can be prevented. One of the non-adherent dressings is impregnated with silicone or paraffin in order to enhance nonadherence [71]. This type of contact layer adheres to dry skin while remaining non-adherent to the wound place, ensuring a painless removal and a reduced risk of damage to the wound site upon dressing change. All these primary wound contact layer dressings focus on the issues of pain and trauma associated with the adherence [71,159].

In work published in 2016, Unnithan et al. [160] fabricated zwitterionic poly(carboxybetaine- co-methyl methacrylate) (CBMA) nanofibers using the electrospinning technique and utilized them as non-adherent and easily removable wound dressings. The CBMA nanofiber membrane showed higher resistance to cell attachment, platelet adhesion, and enhanced antibacterial activity. The inert cell property of nano-fibrous mats gives a lot of advantages for wound care management, and such non-cell adherent wound dressing membranes should be used as easily detachable, painless bandages. These membranes will not provoke any pain upon frequent change. Further, the healing tissue will not be damaged since the newly formed layer of skin is not disturbed. A minimal scar can be expected when this type of wound dressings is used.

In order to decrease the wound adherence of commercial silver-based wound dressings without compromising the antimicrobial activity, Asghari et al. [161] deposited a non-adherent polyacrylamide (PAM) hydrogel layer on two types of dressings. They demonstrated that the PAM layer significantly reduced the adherence of commercial antimicrobial dressings in an in vitro gelatin model, at the same time, preserving the antimicrobial efficacy and decreasing their cytotoxicity.

Rajalekshmi et al. [162] prepared silver nanoparticle incorporated gelatin-hydroxypropyl methacrylate hydrogels for non-adherent wound dressing applications. Due to the antimicrobial properties, the hydrogel showed inhibitory activity against *S. aureus*. Also, in vitro cell, culture tests proved the absence of cytotoxicity and nonadherence character to dermal fibroblasts.

Naseri-Nosar et al. [82] prepared cerium oxide nanoparticle-containing poly (ε-caprolactone)/gelatin electrospun film as a potential wound dressing material. The results provided evidence supporting the possible applicability of CeO_2_ nanoparticle-containing wound dressing for successful wound treatment. Also, the obtained wound dressings proved to present almost the same values of adhesiveness of the wound, like the commercial non-adhesive wound dressings Biatain and Allevyn produced the companies Coloplast (Humlebaek, Danmark) and Smith & Nephew (London, UK), respectively.

Adherence of wound dressings to the wound can delay the wound healing process and be extremely distressing to the patient. Perhaps not surprisingly, pain-free removal and nonadherence are considered to be the essential features of a dressing. The preferable characteristics of these products also include conformability to the wound bed, transmission of wound exudate to the secondary dressing, the ability to stay in situ over wear time, and ease of use [163].

### 2.7. Multilayer Dressings

Multilayered dressings are mostly a combination of the previously described dressings. There is a combination of a semi- or non-adherent layer and a highly absorptive layer of fibers, such as rayon fabric, cotton, and others, that is usually applied for lacerations, burns, abrasions, or surgical incisions [164,165]. Furthermore, a combination of hydrocolloids and alginates was applied for the treatment of burns, superficial leg ulcers, and pressure wounds. For the treatment of chronic wounds, there was fused the hydrogel, foam, and polyurethane layers into a multilayered dressing [71,166]. Multilayer hydrogels consisting of more than two layers are designed to meet the requirements of wound healing by choice of chemical and physical properties of the constituent biomaterials. Multilayer hydrogel wound dressings combine the advantages of each of the constituents. Multilayer hydrogels consisting of a drug-loaded layer allow for the controlled drug release over a prolonged time by maintaining mass transfer limitations for drug molecules throughout the polymeric matrix [4,165,167].

The layer-by-layer (LBL) approach can be used to a comprehensive range of polymers enabling the design of functional biomaterials with desired properties without any additional chemicals [168]. The development of multilayer hydrogels using the LBL technique depends on the adsorption of the polymers having oppositely charged groups layer by layer, providing the incorporation of them into multilayers [168,169]. Multilayer hydrogels fabricated by the LBL self-assembly method comprised of various polymeric layers have gained a great interest in drug delivery applications owing to their facile preparation procedure, tunable morphological features, and high biocompatibility providing the drug molecules released from the matrix in a controlled manner [4,170]. Zhou et al. [171] obtained antibacterial multilayer films by LBL immobilizing lysozyme and gold nanoparticles on nanofibers. The result of the microbial inhibition test showed that the composite nano-fibrous mats present high antibacterial activity against *E.* *coli* and *S.* *aureus*, which could be used for wound dressing applications.

Multilayer wound dressings providing a better healing process than single layer dressings have become promising alternatives [172]. In a recent study, Tamahkar et al. [4] designed a novel multilayer hydrogel wound dressing for antibiotic release composed of natural polymers with a water-based approach for effective wound healing. Multilayer hydrogels were prepared via LBL self-assembly through electrostatic interactions between four polymeric layers using carboxylated polyvinyl alcohol, gelatin, hyaluronic acid, and again gelatin. Multilayer hydrogels loaded with ampicillin showed antibiotic release for 7 days. Also, the dressings proved antibacterial activity against oxacillin sensitive *S. aureus* and showed no toxic effect on cultured fibroblasts, indicating an effective option for selective treatment of bacterial infections. Another novel biocompatible multilayered nano-fibrous dressing was fabricated by Shokrollahi et al. [173] in 2020, composed of poly(εcaprolactone) (PCL) nanofibers as the first layer, hybrid nanofibers of chamomile/carboxyethyl chitosan (CECS)/PVA and PCL as the second layer, and chamomile loaded CECS/PVA as the third layer using electrospinning. 

Dressing-based nanofibers showed an improved antibacterial effect than the commercial wound dressing Ag coating. Overall, based on this study, 20 wt. % chamomile loaded mat should be appropriate for wound healing application, due to its antioxidant, antibacterial, biocompatibility, and mechanical properties. The interest in applying soft nano-fibrous patches with high surface to volume ratios as carriers for plant extract delivery has been increasing.

Although there have been remarkable improvements in the production of wound dressings, the evolution of novel antibacterial multilayer wound dressings with cost-efficiency, transparency, and high healing capacity is still of interest [4,170]. However, the multilayer foam dressings are successfully applied in the prevention of pressure ulcers [41].

## 3. Conclusions

Wound management is an essential and increasing issue worldwide. Knowledge of wound dressing available products and clinical expertise regarding wound dressing selection are two significant aspects in wound management to guarantee evidence-based wound care.

Existing approaches to modern wound management are focused on providing a moist wound environment. This aspect is beneficial for tissue remodeling (re-epithelialization) owing to good control of the wounds’ humidity, together with improved pH control of the wounds and superior oxygen permeability and concentration in the wounds. Additionally, features like biodegradability, biocompatibility, fluid absorption, and antioxidant ability of the bioactive dressing materials are essential for improved wound healing.

Advanced wound dressings are divided into seven categories: semi-permeable films, semipermeable foam dressings, hydrogel dressings, hydrocolloid dressings, alginate dressings, non-adherent contact layer dressings, and multilayer dressings (Table 2).

Distinguishing the properties of conventional materials and new nanomaterials in wound-healing therapy may bring up in managing complex wounds, like chronic and ischemic ulcers, by a combination of nano and/or micro-hybrid materials. It is essential to accomplish further preclinical studies in order to include the benefits of nanoparticles in tissue regeneration. Further research needs to be performed to identify new nanomaterials and their performances in accelerating chronic wound healing. Development of new biocompatible and biodegradable nanomaterials, which are capable of regulating all phases of wound healing, incorporating the antibacterial property, self-healing property, excellent mechanical properties, and adhesion into the wound dressings to improve its performance in clinical applications may be future scope for researchers working in this area.

## Figures and Tables

**Table 1:** Studies concerning polyurethane-based semipermeable dressing foams.

Materials	Important Aspects	Results	Application	References
Usnic acid-loaded polyaniline/polyurethane	It is essential to select an optimal doping level for usnic acid; antibiofilm character due to the specific surface for polymer adhesion; improved anti-biofilm action provided by usnic acid;	antibiofilm improved action provided by usnic acid; biofilm inhibition and reduction in the viable bacterial population; low cost; eco-friendly;	Against *E. coli* and *S. aureus* in wound dressing	Marcelo R. dos Santos et al., 2018 [98]
Polyurethane, biomacromolecule, and asiaticoside (AS)	Polyurethane combined (PUC) foam dressings with various biomacromolecules obtained with the adsorption of asiaticoside and silver nanoparticles; biomacromolecules had varying effects on physicochemical and mechanical properties of PU foam; carboxy- methylcellulose (CMC) had the highest compression strength but the lowest water vapor transmission; high water absorption was obtained for foams with CMC, alginate, hydroxypropyl methylcellulose and low molecular weight chitosan; concentrations up to 12% had more prominent effect;	On healthy volunteers: the prepared foam dressing caused no skin irritation and retained moisture comparable to the commercial product; in patients with traumatic dermal wounds: healing improvement with shorter wound closure time, higher reepithelialization and less pain score was from the selected foam dressing compared to standard gauze soaked with chlorhexidine;	Traumatic dermal wound treatment	Namviriyachotea et al., 2019 [99]
Polyurethane, silver, and asiaticoside (AS)	Foam dressing containing natural polyols, silver nanoparticles, and AS; hydroxypropyl methylcellulose, chitosan, and sodium alginate were individually mixed with the main polyols, polypropylene glycol, in the formulation while the active components were impregnated into the obtained foam dressing sheets;	Antimicrobial effect; non-cytotoxicity; type and amount of the natural polyols slightly affected the pore size alginate and hydroxypropyl methylcellulose improved water sorption-desorption profile and compression strength; need further investigation;	Dermal wounds	Namviriyachotea et al., 2019 [100]
PU-silica hybrid foams	Hybridization of bioactive silica nanoparticles with PU;	Enhanced biocompatibility and wound healing capabilities	Wound healing	Song et al., 2017 [93]
Polyurethane/ZnO nanocomposite foams	Induced phase separation method;	Appropriate morphology to keep the proper environment at the wound/dressing interface; low cytotoxic potential; effective against Gram-positive and gram-negative bacteria	Wound healing	Bužarovska et al., 2019 [95]

**Table 2:** Overview of most used advanced wound dressings.

Wound Dressings	Materials	Shape/ Form	Application	Advantages and Properties	Disadvantages and Limitations
***Semi-permeable film dressings***	Non-porous polyvinyl polymer; transparent and adherent polyurethane coated with an adhesive layer [38,41,63,64,66,71,174]	Films	Superficial wounds as primary dressings [63,64], or as secondary dressings when used in combination with foam dressings in more substantial exuding wounds; epithelializing wound, superficial wound and shallow wound with low exudates [41,66]; superficial burns; minor abrasions and laceration; radiation dermatitis; postoperative sutured wounds; prevention of pressure injuries [38,71]	Thin and semipermeable transparent film, highly elastic and flexible [2,70]; can conform to any shape (patient‘s body) [63,64]; do not require additional tapping; maintains moist and protective environment; prevents bacterial migration and provide a barrier to external contamination; impermeable to liquids and bacteria; provide no cushioning allow inspection without dressing removal (transparent) [38,71]; can remain in place for 1 week; adhere to healthy skin but not to wound [65].	Not suitable for exuding wounds (moderately to highly), may not prevent maceration [41,66]; not used in the management of infected wounds; may damage fragile skin [38,71]
***Semi-permeable foam dressings***	Polyurethane [63,71], polyaniline [98]	Foam	Infected ulcers; pressure ulcers; venous ulcers; moderate to heavily exuding wounds [92]; superficial and cavity wounds; skin tears; skin grafts and donor sites [38,71]	Absorbent (absorbency can be controlled by the foam’s thickness, texture and pore size); provides moist interface; good absorbent; can present both hydrophobic or hydrophilic properties [63,71]; should be changed once saturated with exudate (range from once daily to once or twice weekly) [63]	Not suitable for dry wounds, necrotic wounds, hard eschar and wounds requiring frequent review [38,71]; may need a retention product; special care needed for patients with fragile skin for nonsilicone types [38]
***Hydrogel dressings***	Hydrophilic, inflatable, and insoluble materials [129]; cross-linked polymers (cellulose, starch or other derived polysaccharides) [37,71,105,106,107,108]	Shapes of sheet hydrogel, amorphous gel, and impregnated gauze [37,105,106,107,108,129]	Wounds with low exudate; dehydrated wounds; burns; surgical wounds, skin tears, and pressure ulcers; grazes/lacerations; radiation oncology burns; donor sites; healing of the painful wounds [37,38,41,71,105,106,107,108,129]	Transparent; absorption of a large number of ulcers; creation of a damp environment that removes dead tissues [110] and foreign materials from the wound; soothing and cooling effects on the skin; facilitate autolytic debridement [63]; antibacterial action [38,129]; should be changed generally every 1 - 3 days; useful in flat wounds, cavities, and sinuses [63,115]	Suitable only for the surface of wounds [129]; can cause maceration in heavily exuding wounds [63]; not suitable for dry wounds or wounds with hardened eschar; not recommended in the management of wounds with anaerobic infections; need secondary dressing [41,71]
***Hydrocolloid dressing***	A mixture of colloidal materials with elastomers and alginates; sodium carboxymethyl cellulose, pectin, gelatin and polyisobutylene [71,129]	Thin films and sheets, or composite dressings [134,135,136,137]	Are the most widely used dressings: surface ulcers [134,135,136,137], minor burns, shock injuries, bruises, acute and chronic wounds, flat wounds, cavities, sinuses, undermining wounds [71,108,127]	Occlusive; prevent water, bacteria, and oxygen from entering into the wound; biodegradable and biocompatible; can absorb minimal to moderate amount of wound fluids; occlusive; reduce the ph of the wound; facilitate inhibiting bacteria growth; provides a moist, hypoxic wound environment [71,129]; day-to-day changes early in the treatment course, with a decrease to every 3 days to 1 week over time, good in “difficult” areas—heel, elbow, sacrum [63,133]	Not appropriate for deeper wounds, especially wounds with an infection and diabetic foot ulceration; does not prevent maceration in heavily exuding wounds; prevent water vapor exchange [38,71,129]
***Alginate dressing***	Calcium salt and sodium alginic acid [129]; polymer extracted From seaweed [71]	Wafers [151,152,153,169], foams [2,170,171], gauzes [172,173,174], fibers [175,176,177]; sheet form; ribbons and ropes [63];	All wound types with high exudate, infected and noninfected wounds; burn wounds [129,144,148]	Good absorbent (absorption of excess wound secretions up to about twenty times of their weight due to high porosity and nonsticky); serializable; useful in cavities and sinuses, and for undermining wounds; need to be changed daily [71,129]	Not suitable for the dried wounds [71,129]; Need a secondary dressing [129,144,148];
***Non-adherent Contact Layer Dressings***	Polyamide, polyethylene, polyethylene terephthalate, can be coated with silicone [71,158,159]	Layers [71,158,159]	Suitable for a wide range of wound types [71,158,159]	Atraumatic removal with nonadherence to the wound site can be left for up to 14 days; used for the protection of newly formed tissue [158,159]	Can be used only in conjunction with a secondary absorbent dressing [71,158,159]
***Multilayered dressings***	Combination of a semi- or non-adherent layer and a highly absorptive layer [4,71,172]	Layers [71,164,165]	Burns, surgical incisions, lacerations, abrasions [71,172]	Possibility to combine priorities of more dressings depending on the combination of used materials [71,164,165]	The thickness of dressing in the case of the use of more voluminous materials depends on the combination of used materials [71,172]
